# TILLING by Sequencing: A Successful Approach to Identify Rare Alleles in Soybean Populations

**DOI:** 10.3390/genes10121003

**Published:** 2019-12-03

**Authors:** Rima Thapa, Militza Carrero-Colón, Katy M. Rainey, Karen Hudson

**Affiliations:** 1 Department of Agronomy, Purdue University, 915 West State Street, West Lafayette, IN 47907, USA; 2 USDA-ARS Crop Production and Pest Control Research Unit, 915 West State Street, West Lafayette, IN 47907, USA

**Keywords:** soybean, raffinose, stachyose, TILLING, *Glycine max*, RFO

## Abstract

Soybean seeds produce valuable protein that is a major component of livestock feed. However, soybean seeds also contain the anti-nutritional raffinose family oligosaccharides (RFOs) raffinose and stachyose, which are not digestible by non-ruminant animals. This requires the proportion of soybean meal in the feed to be limited, or risk affecting animal growth rate or overall health. While reducing RFOs in soybean seed has been a goal of soybean breeding, efforts are constrained by low genetic variability for carbohydrate traits and the difficulty in identifying these within the soybean germplasm. We used reverse genetics Targeting Induced Local Lesions in Genomes (TILLING)-by-sequencing approach to identify a damaging polymorphism that results in a missense mutation in a conserved region of the *RAFFINOSE SYNTHASE3* gene. We demonstrate that this mutation, when combined as a double mutant with a previously characterized mutation in the *RAFFINOSE SYNTHASE2* gene, eliminates nearly 90% of the RFOs in soybean seed as a proportion of the total seeds carbohydrates, and results in increased levels of sucrose. This represents a proof of concept for TILLING by sequencing in soybean.

## 1. Introduction

Soybean provides 70% of the global supply of protein meal [1]. However, the soybean seed also contains carbohydrates that are considered antinutritional. Raffinose and stachyose family oligosaccharides (RFOs) are not easily digested by monogastric animals [2,3], and studies have suggested that lower levels of raffinose and stachyose contribute to faster livestock growth and to better overall health [4,5]. 

Overall, the identification of genetic variation in the carbohydrate biosynthesis pathway of soybean presents a good opportunity for optimizing meal composition for soybean consumers. There are several genes in the carbohydrate biosynthetic pathway that have previously been used to reduced raffinose and stachyose levels, and related genes whose functions are unexplored. The soybean genome contains four genes encoding raffinose synthase enzymes. *RAFFINOSE SYNTHASE2* (*RS2*), *RAFFINOSE SYNTHASE3* (*RS3*), and *RAFFINOSE SYNTHASE4* (*RS4*) share 60% amino acid identity with the *Arabidopsis* raffinose synthase enzyme, and soybean *RAFFINOSE SYNTHASE1* (*RS1*) is less similar [6]. A naturally-occurring polymorphism in the *RAFFINOSE SYNTHASE2* gene was discovered in the soybean accession PI 200508, a deletion of three nucleotides relative to the reference sequence that results in the loss of tryptophan 331 (W331-) in the protein sequence, and has the phenotypic effect of increasing the ratio of sucrose to RFOs in the seed [6]. Reverse genetics approaches also previously identified a missense mutation (T107I) in the *RS2* gene that reduces levels of raffinose and stachyose and increases sucrose levels [7]. Both of these *rs2* mutants have reduced levels of raffinose (0.1–0.2% raffinose as a percentage of total seed carbohydrates) relative to lines wild type for *RS2* (0.8–1% raffinose) in replicated field tests [8]. Stachyose levels are also reduced in the *rs2* single mutant (0.5–2% of total seed carbohydrates) contrasted with 4% of total carbohydrate in wild type lines, and moderate increases in total sucrose levels [8,9]. A biotechnological approach silencing the *RS2* gene in soybean seeds also achieved significant reductions in seed RFOs and an increase in sucrose [4]. Mutation in *RAFFINOSE SYNTHASE3* has been shown to reduce RFOs further when combined with a non-functional allele of *rs2*, resulting in ultra-low levels of raffinose and stachyose of less than 2% as a fraction of total seed carbohydrates [8,9,10,11]. While the decrease in RFO content is desirable for meal digestibility, the alteration of carbohydrate partitioning resulting in increased levels of sucrose, which contributes to the metabolizable energy in soybean meal is an added advantage [12,13]. RFOs have been demonstrated to have a role in desiccation and cold tolerance in plants and may contribute to seed vigor [14,15,16,17,18,19]. However, studies have shown that soybean *rs2* and *rs3* mutants germinate and emerge with normal efficiency [11,20]. Overall, variation for RFO content is limited in available soybean germplasm [21], and it remains an open question in the field of soybean improvement what impact a null mutation in *RAFFINOSE SYNTHASE* could have on seed carbohydrate levels, and which genetic combination optimally reduces RFOs across growing environments to optimize meal traits.

The *Cel I* endonuclease-based TILLING (Targeting Induced Local Lesions in Genomes) approach has previously been successfully implemented in soybean and resulted in the identification of mutants in seed composition, disease signaling, and other important agronomic traits [7,22,23,24,25]. The reverse-genetic method TILLING presents the ability to identify mutants without prior knowledge of the phenotype or the degree of phenotypic severity. Further benefits to TILLING are the capability to identify mutations that may be lethal or result in reduced viability in the homozygous state. Particularly for the soybean genome, where most genes have two highly similar homeologs, which are both functional. Another advantage that TILLING provides over phenotypic screens is a means to identify mutations in genes where deleterious effects can be masked by the presence of another gene that compensates for the loss of function. Additionally, the resulting mutations are non-transgenic variation and can be used in both conventional or transgenic breeding programs. However, there are several significant challenges: Each gene is different in its coding sequence and GC content and in turn the probability of creating an amino acid change or nonsense mutation as a result of a single base pair change. In many cases, DNA point mutations can be silent or create synonymous changes that are unlikely to be deleterious to the protein. Another challenge is the cost: While sequencing costs per base have declined precipitously in recent years, the effort of maintaining and extracting DNA from large populations, library construction, barcoding, and high-fidelity amplification, as well as the technical effort to perform these techniques, remain a cost impediment. Finally, soybean has undergone a relatively recent genome duplication, and homologous genes are, in some cases, identical at the coding level, presenting a challenge in detecting mutations in one specific member of the homologous pair [26]. In the case of genes involved in carbohydrate biosynthesis, a phenotypic screen of a large population is tedious and costly and requires HPLC (high performance liquid chromatography) analysis of individual samples. In the case of the *RAFFINOSE SYNTHASE* gene family, it is known that *RS2* and *RS3* both contribute to carbohydrate partitioning. We sought to obtain additional and more severe mutant alleles to further reduce raffinose and stachyose levels in soybean seeds. We pursued a reverse genetic strategy utilizing high throughput sequencing (TILLING-by-Sequencing, TbyS [27]) to identify additional loss-of-function mutations in the *RS2* and *RS3* genes. To reduce costs, we constructed only one-dimensional pools, which limited the number of libraries. After sequence analysis, we selected mutations that we expected to be deleterious. As a secondary screen, we designed PCR-based SNP (Single Nucleotide Polymorphism) markers to confirm the polymorphisms and determine which pool and subpool contained the mutant individual. This provided an alternative method of confirming the polymorphism, as well as a usable marker for downstream breeding approaches.

## 2. Materials and Methods 

### 2.1. Plant Material and Growth Conditions

Mutagenesis of soybean (*Glycine max*) cultivar Williams-82 with N-nitroso-N-methylurea (NMU), as previously described [22,28]. M_1_ and M_2_ plants were grown in the field in West Lafayette, Indiana, and leaf disc punches were obtained from each M_2_ plant. For validation of phenotype, crosses to a mutant containing the *rs2* W331-allele (line KB10-23#1677) were performed in the field, and F_1_ plants were advanced and genotyped in the greenhouse. F_2_ populations were planted in the field during the subsequent growing season, and individuals were genotyped to select double and single mutants and wild type siblings [29]. PCR based genotyping was performed using CAPs or dCAPS markers specific for *rs2* and *rs3* mutant alleles (listed in Supplemental Table S1).

### 2.2. DNA Extraction, Amplification, Sequencing, and Mutant Identification

DNA was extracted from 100 mg freeze-dried leaf tissue using the Omega EZ 96 Plant DNA kit (Omega Bio-tek, Norcross, GA, USA). Samples were quantified by visualization on agarose gels and digital densitometry with ImageQuant software (GE Healthcare) and diluted to a concentration of 9 ng/μL. These individual samples were diluted to a working concentration of 3–6 ng/μL in 8-sample pools. PCR primers (listed in Supplemental Table S1) were designed to amplify exons of the *RS2* (Glyma.06g179200) and *RS3* (Glyma.05g003900) genes. We chose to pursue amplification of exon 1 of the *RS2* and *RS3* gene for the possibility of the introduction of an early termination codon and because of the reliable and robust amplification observed from these primer sets. Amplification was performed (95 °C for 60 s, 5 cycles of 94 °C 30 s, 54 °C 20 s, 68 °C for 180 s, the 25 cycles of 94 °C 30 s, 56 °C 20 s, 68 °C for 180 s) on DNA sub-pools (8 samples) using the Advantage 2 high fidelity polymerase mix (Takara Biosciences). Subpool amplicons were combined into larger pools of 512 samples, purified using Ampure (Beckman Coulter, Indianapolis, IN, USA), quantified using fluorimetry on the Qubit 2.0 (Life Technologies, Carlsbad, CA, USA). In addition, 1.5 μg of each sample was sheared into 200 bp fragments, and Illumina libraries were constructed using standard protocols as described previously [29]. Three libraries were prepared for the *RS2* amplicon, and 3 libraries were prepared for the *RS3* amplicon. Each library included a non-overlapping set of M_2_ individuals. The *RS2* and *RS3* amplicons were sequenced for a total of 1533 unique M_2_ individuals. DNA from PI 200508, which carries multiple point mutations in the *RS2* gene was included as a sample in each *RS2* library to act as a positive control for mutation detection. Sequencing was performed on the Illumina HiSeq2500, to obtain paired-end reads of 150 bp.

### 2.3. Sequence Analysis

Illumina sequence assembly was performed with bowtie2 [30] to reference soybean genomic sequences corresponding to our targeted regions from the Williams 82 a2.v1 genomic sequence obtained from Phytozome [31]. The samtools pileup utility was used to identify sequence variants and determine the variant frequency, and output frequency was parsed from the alignment via the mpileup-tools suite (run mpileup.py and mpileup-parser-v2.py) obtained from (http://comailab.genomecenter.ucdavis.edu/index.php/mpileup, last visited 10/2019) as described [27]. Mpileup was used with the following parameters: –d 8000000–q 21–Q 21 (maximum depth per base in each input BAM (binary alignment map) file of 8 million, minimum base, and minimum mapping quality scores of 21). Base frequencies that substantially exceeded background values were targeted as potential induced variation (see Results section). For each predicted mutation identified in the parsed pileup file, a manual comparison with predicted coding sequence was then used to determine if the mutation caused a non-synonymous change in the coding sequence, and SIFT (Sorting Intolerant From Tolerant) was used to estimate the likelihood of a damaging mutation and further select targets for PCR-based mutation screening [32]. Only changes predicted to be both non-synonymous and damaging were pursued for isolation by the secondary screen.

### 2.4. SNP Assays 

For each novel mutation we pursued, a positive control was generated in the form of a plasmid carrying the mutated sequence. First, *RS2* or *RS3* amplicons from wild type Williams-82 were inserted into the TOPO-TA plasmid (Invitrogen, Carlsbad, CA, USA) and validated by Sanger dye-terminator sequencing using standard M13 forward and reverse sequencing primers. Second, selected polymorphisms were introduced into the soybean sequence in the plasmid using the QuikChange Site-Directed Mutagenesis kit (Agilent, Santa Clara, CA, USA) following standard kit protocols. Cleaved amplified polymorphic sequence (CAPS) and derived CAPS (dCAPS) genotyping assays to discriminate the wild-type from the novel mutant sequence were designed using dCAPS Finder [33] and tested on the mutated plasmids, which became positive controls for pool screening. Amplification using mutation discriminating CAPS/dCAPs assays was carried out on pools of 8 M_2_ samples, and finally on individual plants once a pool was found to carry a mutation of interest. These assays were also used to genotype F_1_ and F_2_ plants. 

### 2.5. Carbohydrate Measurement

Crosses were performed to combine mutant *rs2 *(W331-) and *rs3* genes. Single F_3_ seeds produced in the field in the 2017 growing season were genotyped for the 2 genes and analyzed for carbohydrate composition by HPLC. In the 2018 growing season, F_3_ plants were propagated, and bulk seed from single F_4_ plants (5 of each genotype) was analyzed by HPLC. A detailed protocol for HPLC from soybean seed was previously described [10]. Statistical significance was calculated with a two-tailed, type 2 *t*-test between wild type and mutant values, with either 8 single seed samples (for 2017 data) or 5 single plant bulk samples (for 2018).

## 3. Results

### 3.1. Sequencing of RS2 and RS3 Amplicons

A fragment of the *RS2* gene corresponding to exon 1 was amplified from pooled mutant genomic DNA with a sample of DNA from the previously described *rs2* mutant (from the PI 200508 accession) [6] included as a spike-in control, and PCR amplicons were pooled for construction of Illumina libraries. From the sequence data, the approximate coverage of each fragment in each library was calculated. For *RS2* libraries 1, 2, and 3, we obtained 43 million, 53 million, and 45 million paired-end 100 bp reads, respectively, totaling 4.4–5.3 Gb of sequence in each, each with an overall alignment rate > 99%. For *RS3* libraries 1, 2, and 3, we obtained 42 million, 37 million, and 44 million paired ends reads, respectively, totaling 3.8–4.4 Gb each. Coverage was not even across the amplicons, but much higher at the ends of the fragments as expected (Supplemental Figure S1). We estimated that minimum coverage per base per plant (considering 512 plant samples per library) was 2000–3000. In the *RS2* sequence, we found a known polymorphism from PI 200508—several polymorphisms in the *RS2* gene have been described from this accession [6], including a G to C change that occurred 598 bp downstream of the translation start site (720 bp from the start of our amplification primer and the sequence obtained in our experiment) in an area of relatively deep coverage of 4.8 million to 5.9 million. This SNP was present at a frequency of 0.83%, 2.2%, and 0.37% (different fraction of representation for each library) roughly consistent with our estimate. Any single base in a diploid individual should be covered by 0.1% of reads. Small variations in sample concentration and amplification efficiency may be exaggerated through the TbyS procedure, which resulted in the observed range of abundance. However, we found many putative polymorphisms that occurred in approximately 0.1% of the reads at any given reference base and suspected that many might be a result of PCR error (Supplemental Figure S2). Thus, to identify new SNPs in the mutant population, an empirically determined frequency of polymorphism from our spike-in control was used as a starting point. After calling SNPs that appeared in the sequenced DNA, we prioritized screening SNPs that met two criteria: (1) An occurrence above a cutoff of 0.15% (above the noise level but somewhat lower than the expected frequency for a homozygous mutation); (2) that created non-synonymous substitutions or nonsense mutations at a conserved position and were, therefore, predicted to be damaging for protein function. We preferred to select SNPs that resulted in a G to A or C to T change diagnostic of NMU-induced mutation (and less likely to be PCR error), and that was unique to one library (thus not a result of amplification of sequences from a homologous sequence in the soybean genome). However, we screened for both transitions and transversions, when the amino acid change was predicted to be damaging (including mutations near the splicing sites of the *RS3* gene). After successful positive control and genotyping assay design, we pursued five putative polymorphisms in the *RS2* gene, and five putative mutations in the *RS3* gene (Supplemental Table S2).

### 3.2. Polymorphism Validation

CAPS or dCAPS assays were designed to screen the pools for putative polymorphisms (Supplemental Table S1). We targeted five putative mutations in the *RS2* amplicon for secondary screening by dCAPs assay (Supplemental Table S2). It was determined that two of the identified SNPs (L180P and S194N) both originated in one plant sample, and we determined that this sample was, therefore, likely to be a contaminating the non-Williams-82 individual in the original M_1_ population (Supplemental Figure S3). While we chose not to pursue this variation in *RS2*, this finding served as partial validation of the TbyS method. We were unable to validate the other sequence polymorphisms that we selected from the *RS2* amplicon in the population, and we hypothesized that they could be due to PCR-generated errors early in the amplification of the products used for sequencing or in library construction.

**Figure 1:**
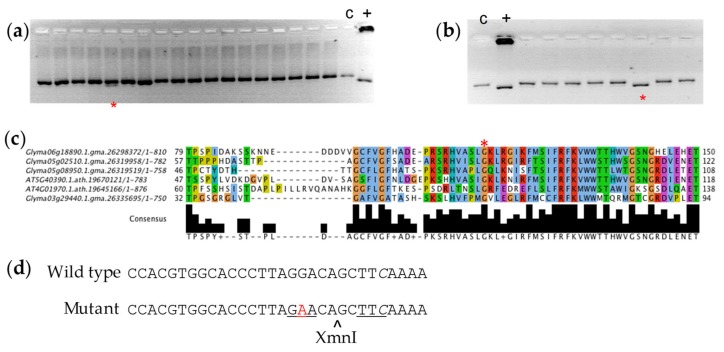
Polymorphism identified in the *RAFFINOSE SYNTHASE3* gene. The presence of multiple bands in (**a**) subpool PCR and (**b**) individual sample PCR (asterisk) after agarose gel electrophoresis indicates single nucleotide polymorphism in *RS3*. c = Wild type genomic DNA control, + = positive control for mutation (see Methods). (**c**) Mutation in *RS3* affects conserved glycine residue at position 75 in the amino acid sequence. (**d**) DNA sequence surrounding the G to A polymorphism (red) in the *rs3_G75E_* mutant. The italicized *C* is where the mismatch in the genotyping primer introduces the XmnI site (underlined) that is cut in the mutant but not the wild-type sequence.

Five putative mutations in the *RS3* amplicon were targeted (Supplemental Table S2), including two sites that were flagged as putatively interfering with splicing. One pool demonstrated a positive band using the confirmatory dCAPS marker for a missense mutation in the *RS3* gene (Figure 1a). It was further determined that this polymorphism existed in a single plant sample (Figure 1b). PCR results suggested that the mutation was homozygous in the M_2_ plant in the original population. A G to A polymorphism 223 base pairs from the translation start site created a glycine to glutamic acid change at position 75 in the predicted amino acid sequence. The glycine 75 residue was conserved across the soybean raffinose synthase family and in *Arabidopsis* raffinose synthase enzymes (Figure 1c). We will refer to this mutation as *rs3_G75E_*. The dCAPs primers and the G to A polymorphism in the mutant introduced a restriction site for the XmnI enzyme, and we used this assay (Figure 1d) developed for screening the pools to identify individuals for phenotypic screening. We were unable to validate and isolate individuals with the other polymorphisms that we observed in the *RS3* amplicon sequence.

### 3.3. Low RFO Phenotype in rs2rs3 Double Mutants

**Figure 2:**
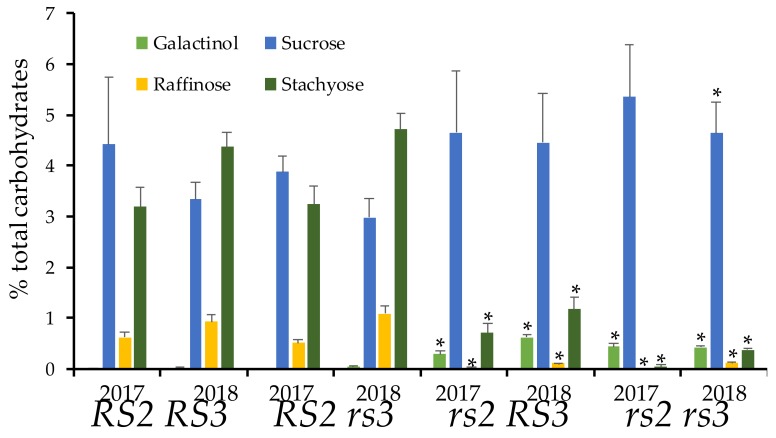
Reduction of raffinose family oligosaccharides (RFOs) in *rs2 rs3* double mutant. Single plants (5 per genotype) were harvested in 2017 and 2018 and assayed for carbohydrate content. Error bars indicate standard deviation, asterisk indicates statistical significance (two-tailed, type 2 *t*-test between wild type and the double or single mutant) at *p* = 0.05.

Based on previous studies with *rs3* mutants, we suspected that it would be difficult to observe a phenotype in the *rs3* single mutant, confirming the expectation on initial analyses for raffinose and stachyose content that showed no effect in the single *rs3_G75E_* mutant. We expected that the *rs3* mutation would, however, enhance the phenotype of *rs2_W331-_* mutants. We crossed *rs3_G75E_* to *rs2_W331-_*, and 11 individuals (to control for variation in the genetic backgrounds of the two parents) were selected from the F_2_ generation of the cross using PCR-based genotyping markers. The seed was harvested from individual *rs2* and *rs3* single mutants, as well as the *rs2rs3* double mutant and wild type siblings. Analysis of seed composition by HPLC in Figure 2 for two field seasons showed that the seeds wild-type for both *RS2* and *RS3* have ~ 4% sucrose, 1% raffinose, and 3% stachyose. Mutation in *RS3* alone has no significant effect on carbohydrate content. As previously observed, the mutation in *RS2* reduced raffinose content to 0.2%, and stachyose levels to less than 1% with a modest increase in sucrose levels. As observed in other studies [10], galactinol was undetectable in lines with the wild type allele of *RS2*. A combination of the *rs2* and *rs3* mutations reduced residual stachyose content, and sucrose increased to 5%. The reductions in raffinose and stachyose were statistically significant in the *rs2rs3* double mutant in both years.

## 4. Discussion

Lower levels of raffinose and stachyose, along with increased levels of sucrose in soybean seed, have been demonstrated to improve the early growth of animals fed on soybean meal rations [4]. While mutation in *RS3* alone does not make a significant difference to raffinose and stachyose levels in the soybean seed, we have shown that in combination with *rs2_W331-_*, *rs3_G75E_* can dramatically reduce levels of raffinose and stachyose, while increasing sucrose levels. The G75E mutation results in a reduction in raffinose and stachyose similar to that of a previously isolated *rs3* mutant allele [10], a variant of the *RS3* sequence that contains two distinct missense mutations that reduce RFOs in combination with a mutant *rs2* allele [11]. Recently, an additional haplotype variant in *rs3* has been demonstrated to have a similar additive phenotype when in combination with *rs2*. While there are distinguishing polymorphisms outside the *rs3* open reading frame in this accession, the mechanism of action of this allele is not understood [34]. As reported here, *rs3_G75E_* is an alternative source that can be used for further non-transgenic improvement in the soybean meal profile, and in the presence of *rs2_W331-_* reduces raffinose and stachyose to levels comparable to these other ultra-low RFO genotypes. Further studies using the combination of *rs2_W331-_* and *rs3_G75E_* will be needed to identify lines where sucrose levels are increased in the double mutants. In this study, genetic loci from the parents controlling physiological maturity were not fixed, and it has been observed that maturity effects contribute significantly to sucrose levels [8]. Interestingly, the roles for *RAFFINOSE SYNTHASE1* and *RAFFINOSE SYNTHASE4* in soybean seed composition remain unknown, and it is untested whether the elimination of these enzymes could further improve the soybean seed carbohydrate profile.

In this case, the reverse genetic TbyS approach enabled the isolation of a new loss-of-function allele of *RAFFINOSE SYNTHASE3* in soybean. We believe that our approach of using CAPS or dCAPS markers to isolate and validate mutant lines represent perhaps the easiest and lowest cost method of this type. The *rs3_G75E_* mutation has no effect on raffinose or stachyose content on its own, and, therefore, could not have been detected using a forward genetics-based phenotypic screen, unless the *rs2* mutation was present in the population before mutagenesis. An advantage to this method is cost: The sequencing-based method of TILLING does not require specific mutation-detection equipment beyond a high throughput sequencing instrument, such as those found in many core facilities. Pooling large numbers of samples into each library reduces the cost of sequencing and library construction. The CAPS/dCAPS screening method is fairly inexpensive and easy to implement in any basic molecular lab, which allows for the inexpensive design of assays for validation of potentially damaging polymorphisms while minimal effort is expended on silent mutations, and the SNP marker can be used effectively in population genotyping. As observed in other TbyS experiments, many sequence polymorphisms [35] were observed in the initial sequencing data, at frequencies that made it difficult to discriminate between false and true positives, and, notably, PCR errors include the same types of nucleotide substitutions as those induced by the mutagen [27,35,36,37,38]. Alternative multi-dimensional pooling strategies (or initial amplification from smaller pools) would circumvent this problem (although with additional costs), and as library construction becomes more routine and less costly, we hope that the TILLING-by-sequencing approach can be used in soybean to identify more mutations with less noise. We are currently applying this successful, straightforward, and inexpensive method to other gene targets.

## Supplementary Materials

The following are available online at https://www.mdpi.com/2073-4425/10/12/1003/s1, Figure S1: Sequence coverage for *RS2* and *RS3* amplicons, Figure S2: Sequence polymorphisms in the *RS2* and *RS3* amplicon sequences, Figure S3: Detection of *RS2* polymorphisms, Table S1: Primer sequences, Table S2: Unique sequence polymorphisms identified in *RS2* and *RS3* amplicons.
