# Supplementary material for: TILLING by Sequencing: A Successful Approach to Identify Rare Alleles in Soybean Populations
Source: Genes (Basel). 2019 Dec 3;10(12):1003. doi: 10.3390/genes10121003 (PMC6947341; doi:10.3390/genes10121003)
Supplement: Supplementary file 1 [file genes-10-01003-s001.zip › genes-641647-supplementary/Figure S1.pdf]

Figure S1. Depth of coverage

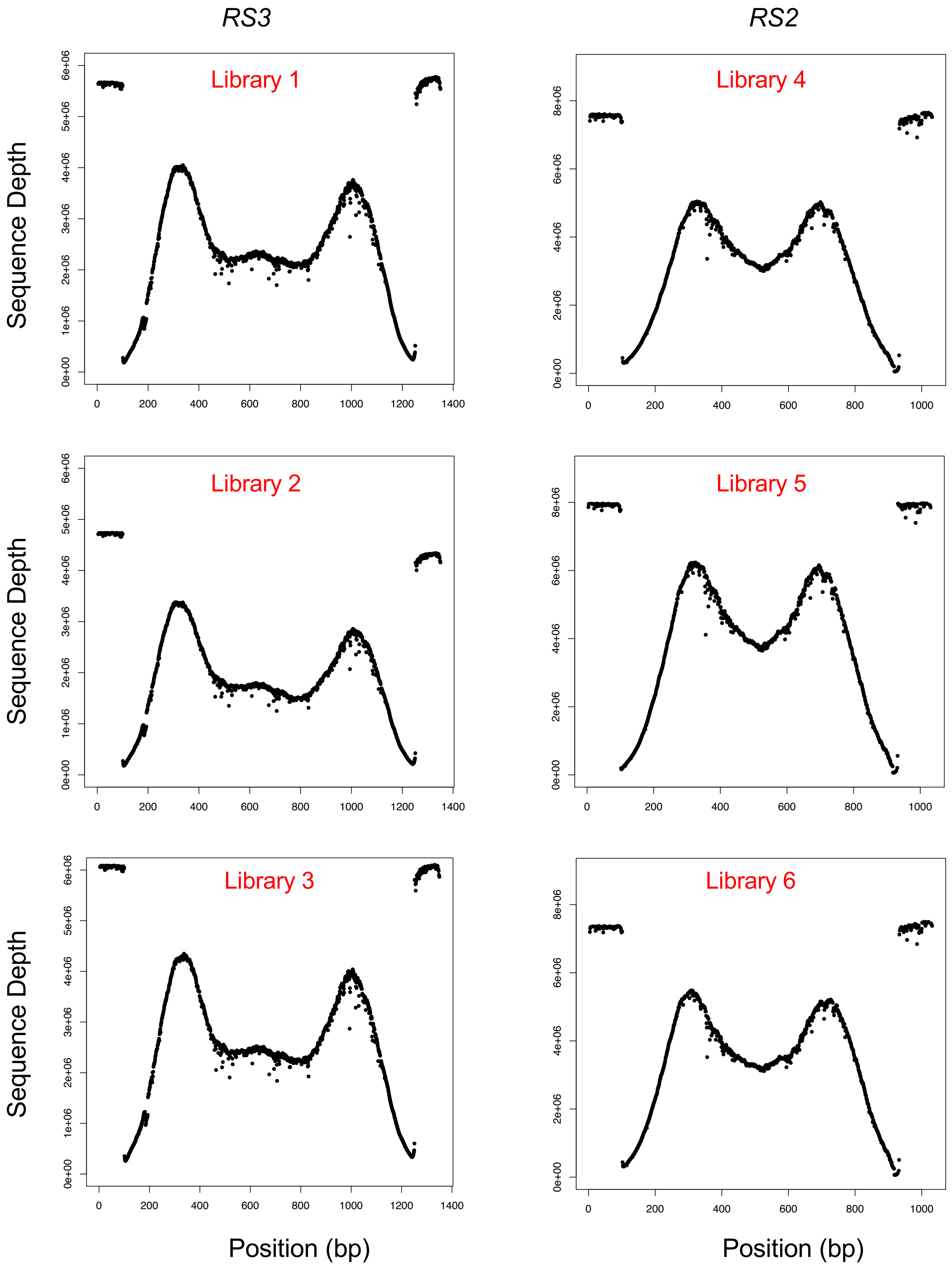

Figure S1. Sequence depth over the *RS2* and *RS3* exon 1 amplicons. Small gap in *RS3* sequence at ~ 200bp into the amplicon is a small inverted repeat that occurs approximately 300bp upstream of the translation start site.
