# Supplementary material for: TILLING by Sequencing: A Successful Approach to Identify Rare Alleles in Soybean Populations
Source: Genes (Basel). 2019 Dec 3;10(12):1003. doi: 10.3390/genes10121003 (PMC6947341; doi:10.3390/genes10121003)
Supplement: Supplementary file 1 [file genes-10-01003-s001.zip › genes-641647-supplementary/Figure S2.pdf]

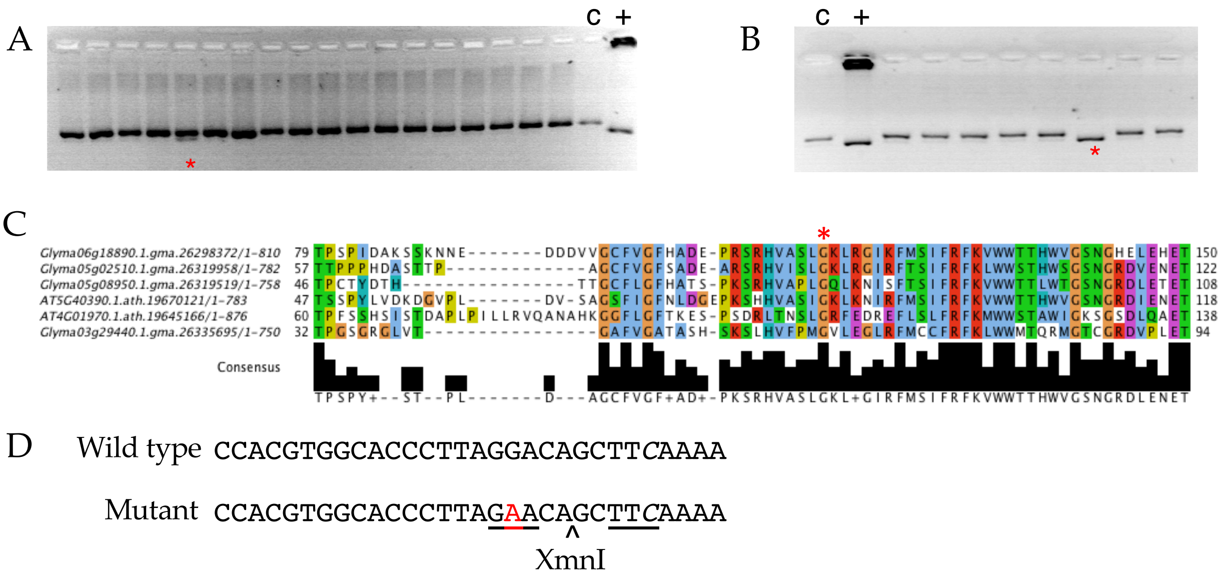

A
c
+
*
B
c
+
*
C
*
Glyma06g18890.1.gma.26298372/1-810 79 TPSPIDAKSSKNNE-------DDDVVGCFVGFHADE-PRSRHVASLGKLRGIKFMSIFRFKVWWTTHWVGSNGHELEHET 150
Glyma05g02510.1.gma.26319958/1-782 57 TTPPPHDASTTP-------------AGCFVGFSADE-ARSRHVISLGKLRGIRFTSIFRFKLWWSTHWSGSNGRDVENET 122
Glyma05g08950.1.gma.26319519/1-758 46 TPCTYDTH----------------TTGCFLGFHATS-PKSRHVAPLGQLKNISFTSIFRFKVWWTTLWTGSNGRDLETET 108
AT5G40390.1.ath.19670121/1-783 47 TSSPYLVDKDGVPL-------DV-SAGSFIGFNLDGEPKSHHVASIGKLKNIRFMSIFRFKVWWTTHWVGSNGRDIENET 118
AT4G01970.1.ath.19645166/1-876 60 TPFSSHSISTDAPLPILLRVQANAHKGGFLGFTKES-PSDRLTNSLGRFEDREFLSLFRFKMWWSTAWIGKSGSDLQAET 138
Glyma03g29440.1.gma.26335695/1-750 32 TPGSGRGLVT----------------GAFVGATASH-SKSLHVFPMGVLEGLRFMCCFRFKLWWMTQRMGTCGRDVPLET 94
Consensus
TPSPY+--ST--PL-------D---AGCFVGF+AD+-PKSRHVASLGKL+GIRFMSIFRFKVWWTTHWVGSNGRDLENET
D
Wild type CCACGTGGCACCCTTAGGACAGCTTCAAAA
Mutant CCACGTGGCACCCTTAGAACAGCTTCAAAA
XmnI

Figure 2.

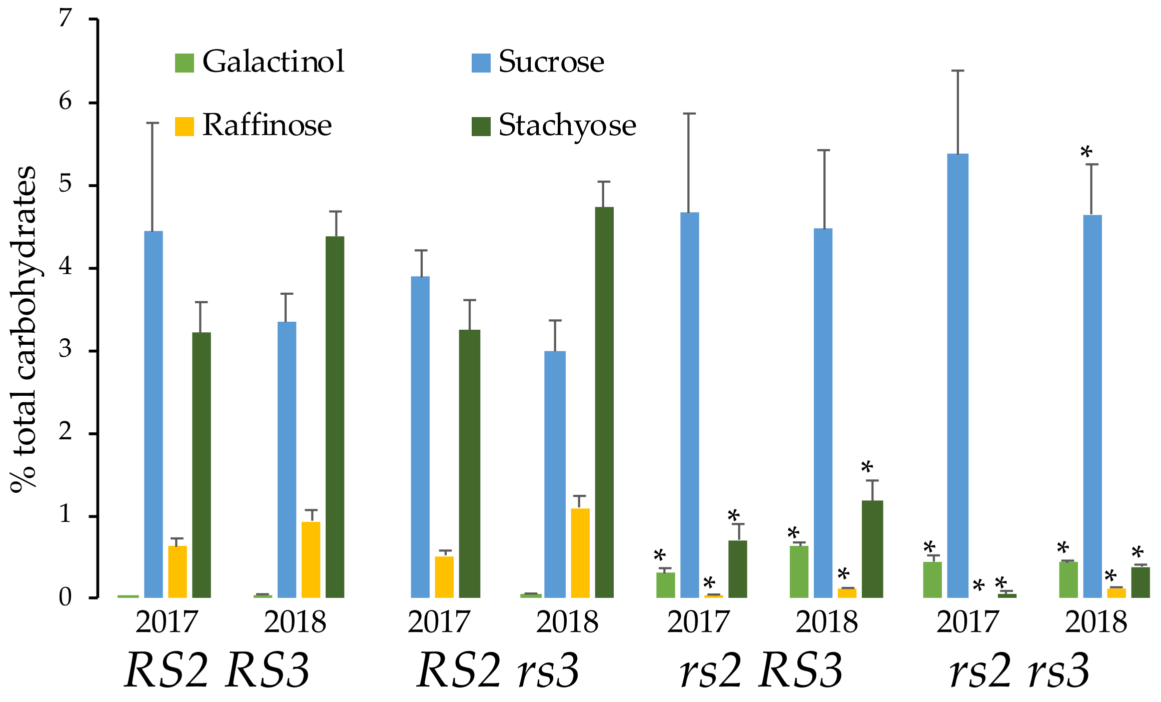
% total carbohydrates
0
1
2
3
4
5
6
7
Galactinol
Sucrose
Raffinose
Stachyose
2017
2018
2017
2018
2017
2018
2017
2018
RS2 RS3
RS2 rs3
rs2 RS3
rs2 rs3

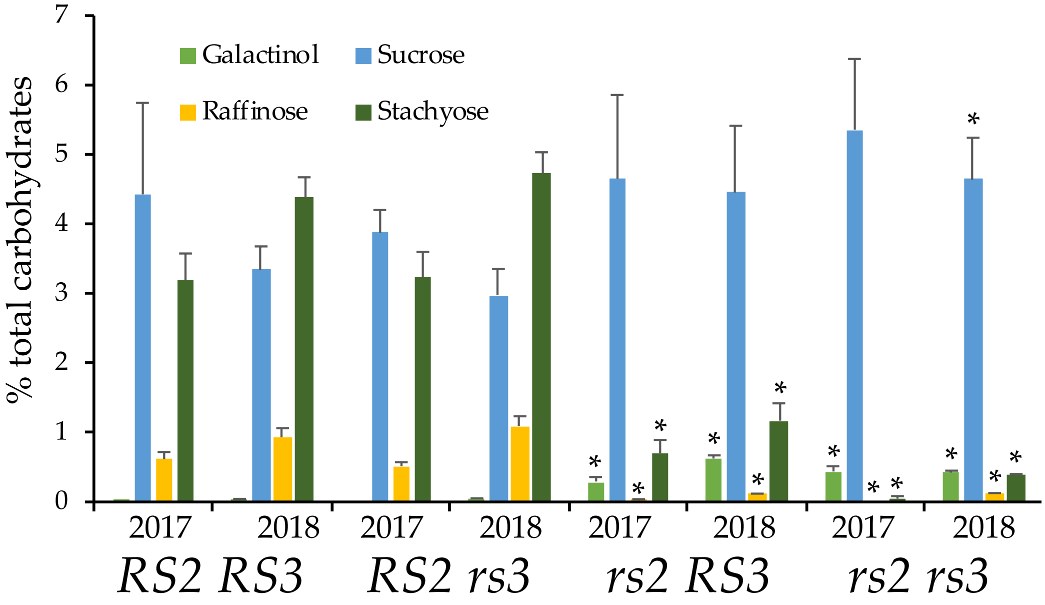

% total carbohydrates
Galactinol
Sucrose
Raffinose
Stachyose
2017
2018
2017
2018
2017
2018
2017
2018
RS2 RS3
RS2 rs3
rs2 RS3
rs2 rs3

Figure S2.

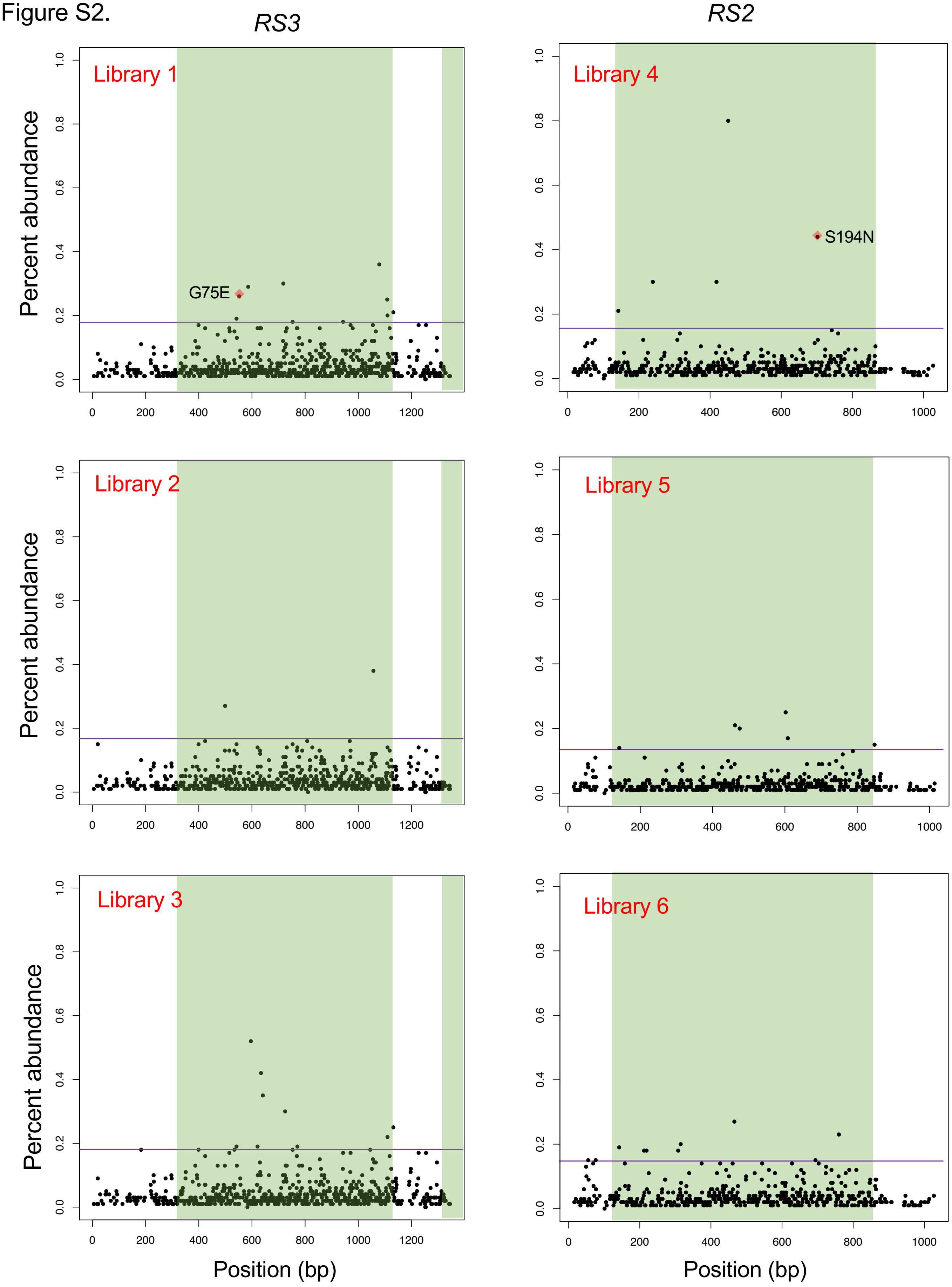

Figure S1. Polymorphisms in the *RS2* and *RS3* amplicon sequences
Abundance of G to A and C to T polymorphisms in *RS2* and *RS3* amplicon sequence in each of three libraries. Green shaded region indicates exon sequence, horizontal line marks a frequency of 0.15 selected as threshold. Highlighted are two validated sequence polymorphisms. Polymorphisms from PI200508 not shown, as these were not G to A or C to T changes.

Figure S3.

***RS2* – L180P**

Pool PCR:

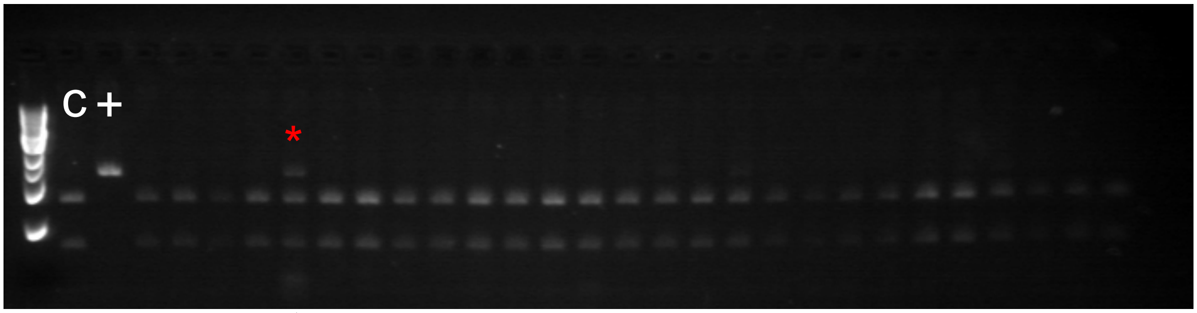

Individual PCR:

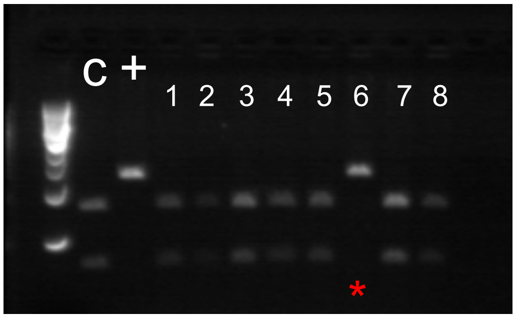

***RS2* – S194N**

Pool PCR:

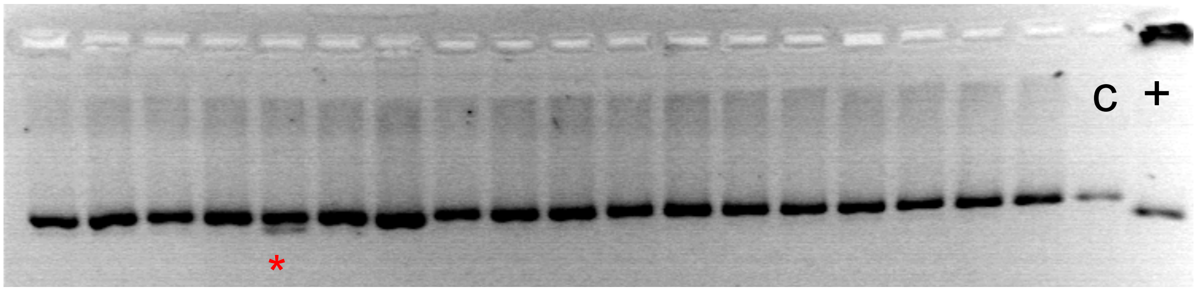

Individual PCR:

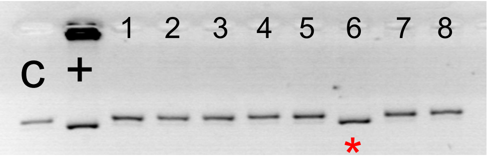

Figure S3. Detection of *RS2* polymorphisms
Pool and subpool PCR showing the appearance of both the L180P and S194N polymorphisms in a single plant sample (plant #6 in pool 5).
