# Supplementary material for: TILLING by Sequencing: A Successful Approach to Identify Rare Alleles in Soybean Populations
Source: Genes (Basel). 2019 Dec 3;10(12):1003. doi: 10.3390/genes10121003 (PMC6947341; doi:10.3390/genes10121003)
Supplement: Supplementary file 1 [file genes-10-01003-s001.zip › genes-641647-supplementary/Figure S3.pdf]

Figure S3.

RS2 – L180P

Pool PCR:

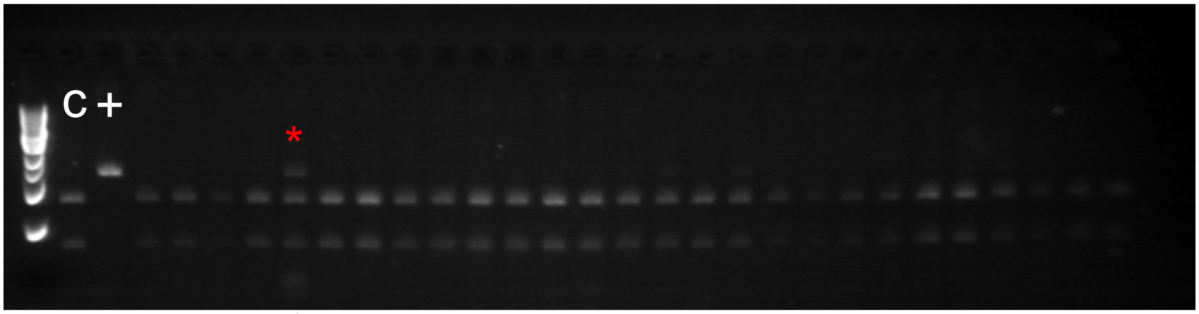

Individual PCR:

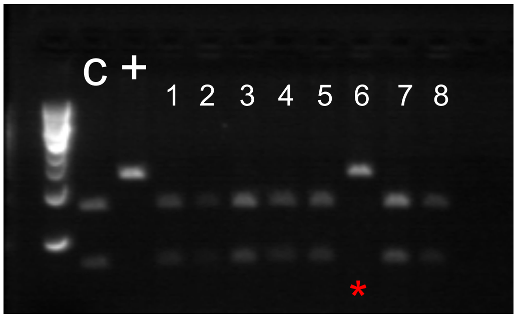

RS2 – S194N

Pool PCR:

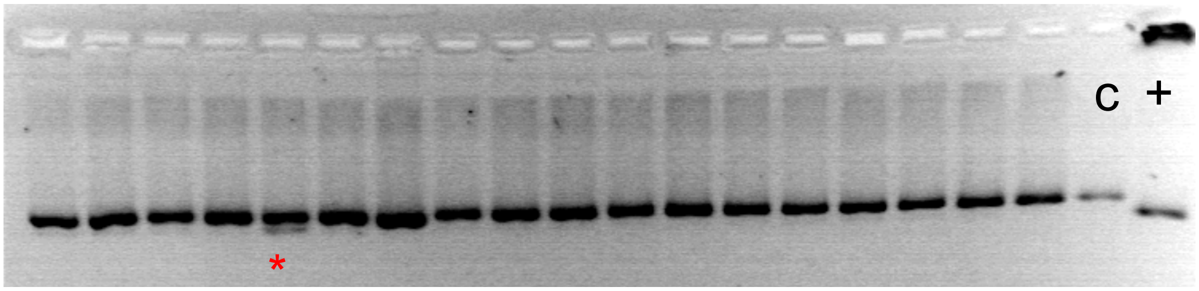

Individual PCR:

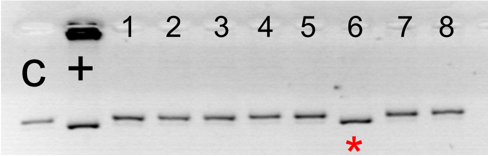

Figure S3. Detection of *RS2* polymorphisms
Pool and subpool PCR showing the appearance of both the L180P and S194N polymorphisms in a single plant sample (plant #6 in pool 5).
